# Perspective: the variable lymphocyte receptor B system in the gut of the sea lamprey

**DOI:** 10.3389/fcimb.2026.1858795

**Published:** 2026-09-07

**Authors:** Alexandra Velthuijzen, Francisco Fontenla-Iglesias, Jonathan P. Rast

**Affiliations:** 1 Department of Pathology and Laboratory Medicine, Emory University School of Medicine, Atlanta, GA, United States; 2 Chemical Biology and Immunology, Leiden Institute of Chemistry, Leiden University, Leiden, Netherlands

**Keywords:** adaptive immunity, agnatha, gut, lamprey, variable lymphocyte receptor

## Abstract

The evolutionary origin of vertebrate adaptive immunity has been a longstanding problem in evolutionary biology. The diversified proteins that orchestrate this complex system are present throughout the jawed vertebrates but are absent in extant jawless vertebrates and invertebrates. From work initially carried out in the sea lamprey (*Petromyzon marinus*) and subsequently extended to other lampreys and hagfishes, we now know that, instead of immunoglobulin domain-based receptors, jawless vertebrates have leucine-rich repeat-based variable lymphocyte receptors (VLRs). Like immunoglobulins (Igs) and T cell receptors (TCRs), these proteins are somatically diversified in lymphocyte-like cells. Of the six VLR loci described in the sea lamprey (VLRA-VLRF), five are expressed exclusively as transmembrane receptors on T-like lymphocytes (VLRA and VLRC-VLRF). Here we focus on VLRB which, like jawed vertebrate immunoglobulins, is expressed both as a cell surface receptor on B-like cells and as a secreted, polyvalent VLRB antibody. Although little is known about the mucosal functions of VLR antibodies, VLRB-expressing cells diversify in gut-associated lymphopoietic tissues and are present in the gut epithelium and other regions of the intestine. We discuss what is known about VLRB and some future directions for understanding its gut-associated functions in relation to what is known in the jawed vertebrates.

## Introduction

The discovery of variable lymphocyte receptors (VLRs) in the sea lamprey (*Petromyzon marinus*) (Pancer et al., 2004) generated a raft of questions, largely through comparisons with jawed vertebrate adaptive immunity. In the ensuing two decades, much has been learned about VLRs and the cells that express them, but many questions persist. There are six germline VLR loci (VLRA-VLRF) in lampreys, five of which are expressed as transmembrane receptors on T-like cells (VLRA, VLRC, VLRD, VLRE, and VLRF) (Das et al., 2025, 2023; Guo et al., 2009; Hirano et al., 2013). The remaining VLR, VLRB, like jawed vertebrate immunoglobulins, is expressed both as a cell surface receptor on B-like cells and as a secreted VLRB antibody (Guo et al., 2009; Herrin et al., 2008).

VLR-expressing cells of all types are present at mucosal sites in the gills, intestine, and skin yet little is known of the functions of the VLRB system in gut immunity. In this Perspective, we review what is known about lamprey VLRB antibodies and the B-like cells that express them with speculative discussion of how future research may explore their functions in gut-associated mucosal immunity.

## Why understanding immunity in lampreys matters

Lampreys and hagfishes (collectively known as cyclostomes) represent the only extant species of jawless vertebrates (agnathans). Their lineage diverged from that of the jawed vertebrates (gnathostomes) approximately 500 million years ago (MYA) (Kuraku and Kuratani, 2006). Notably, the cyclostomes share a genomic history that differs from that of the jawed vertebrates with respect to whole genome duplication events that shaped the genomes of extant species in each lineage (Marlétaz et al., 2024; Yu et al., 2024). The current consensus is that all living vertebrates share a common ancestor that underwent a whole genome duplication event (1R). Shortly after the agnathan-gnathostome divergence, an ancestral gnathostome then underwent a second whole genome duplication (2R) while an ancestral cyclostome ancestor underwent additional independent genome duplications, possibly a hexaploidization. Although this evolutionary history can make it difficult to unambiguously trace gene orthology relationships, it also offers a window into a formative period of vertebrate evolution that coincides with the emergence of vertebrate adaptive immunity.

## The structure of VLRB receptors and antibodies

**Figure 1:**
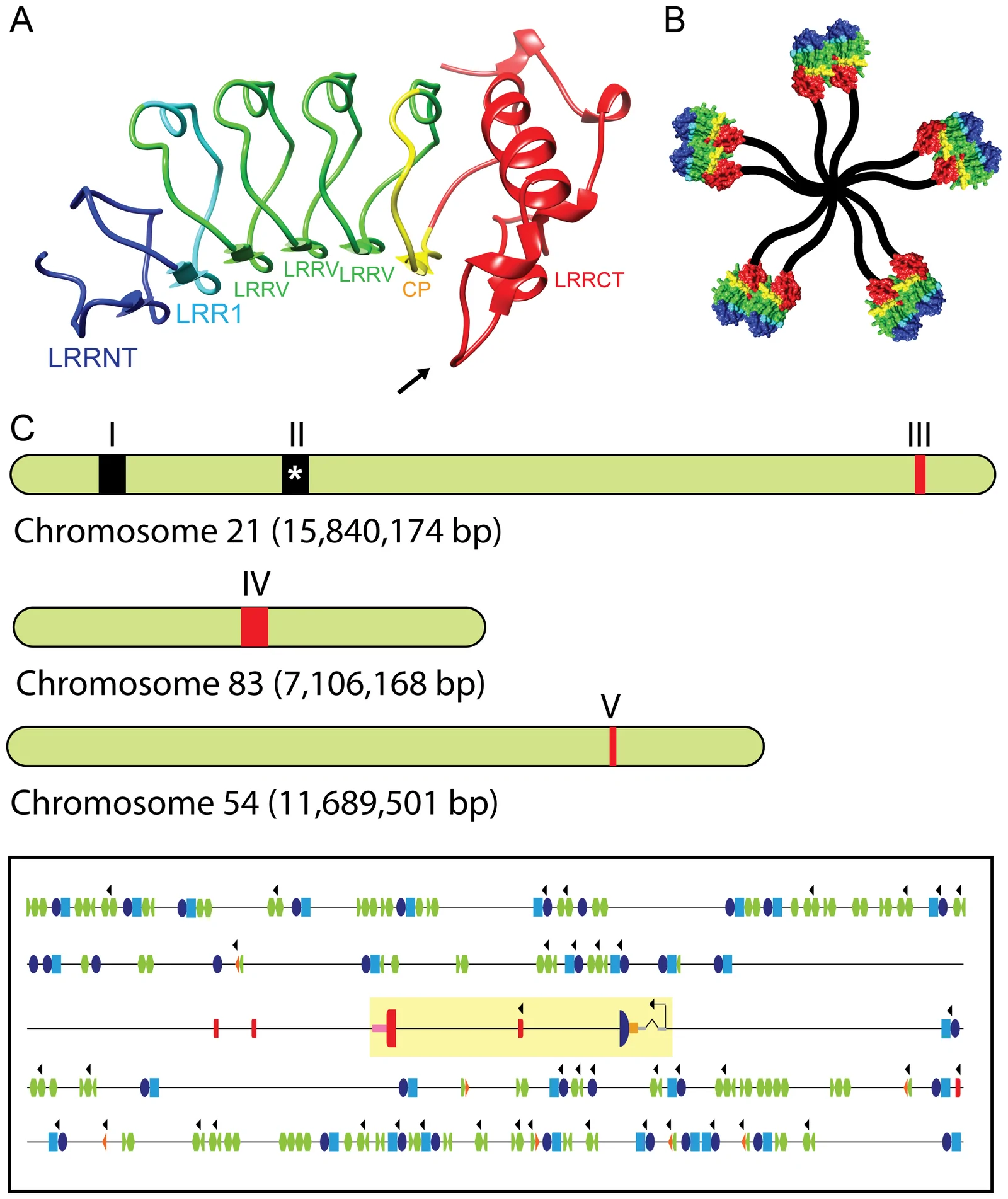
VLRB structure and genomic organization. **(A)** Ribbon model of the LRR region of a representative assembled VLRB (AY577959.1). LRRNT (dark blue), LRR1 (light blue), LRRVs (green), connecting peptide (yellow) and LRRCT (red). The LRRCT loop is indicated with an arrow. **(B)** An illustration of the structure of a soluble VLRB antibody with stalk regions (black lines) linked by disulfide bonds at their C-terminal regions (center). Five VLRB dimers make up the antibody molecule. **(C)** The chromosomal distribution of the five VLRB donor cassette clusters (black and red boxes). Cluster II on chromosome 21 (asterisk) is the location of the germline gene. Clusters that consist entirely of 5′-LRRCT cassettes or 5′-LRRCT/CP cassettes (Cluster IV) are shown as red rectangles. The inset illustrates donor cassette organization in a 300 kb central section of Cluster II surrounding the germline gene. These diagrams are based on analysis of the GCF_010993605.1 genome assembly (Das et al., 2021); the organization shown is largely unchanged in the current reference assembly (GCF_048934315.1).

VLRB monomers have a curved solenoid-like structure composed of leucine-rich repeats (LRRs): an N-terminal LRRNT, an 18 amino acid LRR1, one or more 24 (or less commonly 23) amino acid LRRV modules that each form a loop in the solenoid structure, a 13 amino acid connecting peptide (CP), and a C-terminal LRRCT that, along with LRRNT, caps the hydrophobic core of the structure through disulfide bond-stabilized interactions (**Figure 1A**). This is followed by a flexible, unstructured stalk region rich in threonine and proline residues and a C-terminal region with eight cysteine residues that form covalent bonds linking dimers and multimers of dimers in the VLRB antibody structure (Herrin et al., 2008; Pancer et al., 2004) (**Figure 1B**). VLRB is anchored to the cell membrane via a glycosylphosphatidylinositol (GPI) linkage to a GPI cleavage site in the C-terminal region. Cleavage at this site eliminates the C-terminal 32 amino acids of the VLRB protein containing seven cysteines that contribute to VLRB antibody stability. This leaves a single cysteine that mediates dimer formation. VLRB can be secreted following lymphocyte activation and differentiation into plasmablast-like and plasma-like cells (Alder et al., 2005). Secreted VLRB antibodies typically consist of five disulfide-linked dimers assembled into a multimeric complex (Herrin et al., 2008) (**Figure 1B**).

In solved crystal structures of monomeric VLRB, most of the variable residues that contact antigens are located on the inner concave surface of the LRR structure which is formed by two antiparallel β-strands from LRRNT, one parallel β-strand each from LRR1, the LRRVs modules, and the CP module. The concave β-strand surface of the solenoid contains three rows of variable, outwardly oriented residues responsible for antigen binding (Han et al., 2008). For many VLRB assemblies, the LRRCT region contains a highly variable extended loop structure that also participates in antigen binding. Similar loop structures are also found in VLRA, VLRE, and VLRF (Das et al., 2025). While the differences in individual LRRV elements do not significantly alter the rigid horseshoe-like geometry of the VLRB solenoid, the flexibility of the LRRCT loop plays a crucial role in many solved crystal structures of VLRB-antigen complexes (Han et al., 2008).

## The genomic organization of VLRB

The germline VLRB gene of the sea lamprey is incomplete with a noncoding intervening sequence of ~12 kb interrupting the region encoding the N-terminal signal peptide plus the first five amino acids of LRRNT and a region encoding the C-terminal invariant 20 amino acids of the LRRCT motif and the stalk region. In sea lamprey, a second transcription start site and signal peptide are encoded upstream of the germline gene and a 5′-LRRCT donor cassette is located within the 12 kb intervening region.

A detailed analysis of the sea lamprey genome sequence (Das et al., 2021), performed using the kPetMar1.pri assembly (Timoshevskaya et al., 2023) but largely unchanged in the more recent UKy_Petmar_22M1.pri1.0 assembly, identified a 785 kb region devoid of other identifiable genes surrounding the partial and complete germline VLRB genes. This region contains 178 donor cassettes encoding fragments of LRR sequences. These contribute to the assembly of the mature VLRB genes. Four additional, more distant clusters of donor cassettes, two on the same chromosome as the germline gene cluster (Chromosome 21) and two on separate chromosomes (Chromosome 54 and 83) contribute other donor cassettes making up a total of ~430 cassettes distributed across 1.7 Mb of DNA on three sea lamprey chromosomes (Das et al., 2021).

The cluster containing the germline gene (Cluster II) and an upstream cluster (Cluster I) on the same chromosome contain the majority of donor cassettes (n=345) with representatives of all LRR types covering the entire assembled region (LRRNT, LRRNT-LRR1, LRRV, CP, and 5′-LRRCT). The remaining donor cassette clusters are more specialized with only 5′-LRRCT cassettes (Clusters III and V) or 5′-LRRCT and CP cassettes (Cluster IV). Notably, many of these 5′-LRRCT cassettes carry the hypervariable loop region. In general, the organization of these donor cassette clusters is similar in other lamprey species (Das et al., 2021). A diagram of chromosomal distribution of these donor cassette clusters in the sea lamprey is shown in **Figure 1C**.

## Assembly of mature VLRB

Functional VLRB genes are assembled through an incompletely understood gene conversion-like process in which donor cassette sequences are copied to extend the incomplete N- and C-terminal regions of the germline gene (Alder et al., 2005; Nagawa et al., 2007). Analysis of partially assembled VLRB sequences indicate that these assemblies occur in a stepwise process that can begin at either end of the intervening region of the germline gene (Alder et al., 2005). This process likely depends on the AID/APOBEC-like cytidine deaminase CDA2 (Rogozin et al., 2007). CDA2 is expressed at its highest levels in lymphopoietic tissues and VLRB assembly is absent in animals carrying CRISPR/Cas9-induced CDA2 mutations (Morimoto et al., 2020). Notably, donor cassettes can be incorporated either in their entirety or as partial sequences generating diversity comparable to that of jawed vertebrate immunoglobulins (Das et al., 2021; Rogozin et al., 2007).

## VLRB expressing cells

VLRB is expressed by lymphocyte-like cells that are transcriptionally distinct from those expressing the other lamprey VLRs, as assessed by both RT-qPCR (Guo et al., 2009; Hirano et al., 2013) and single-cell RNA sequencing (scRNA-seq) (Das et al., 2025; Huang et al., 2024). These cells vary in morphology: small lymphocytes predominate in the blood of immunoquiescent animals and, after immune stimulation, larger activated B-like cells and plasma-like cells with greatly expanded rough endoplasmic reticulum are present (Alder et al., 2008). The presence of differentiated plasma-like cells has been known for some time (Zapata et al., 1981) and these are now known to express polyvalent VLRB antibodies (Herrin et al., 2008). Both hybridization chain reaction (HCR) fluorescent *in situ* hybridization and immunohistochemistry identify cells with low and high levels of VLRB expression that may correspond to resting cells and successive stages of differentiation of VLRB antibody-secreting cells.

## VLRB cells in the lamprey gut

In mammals, B cell subsets and Ig isotypes have specialized functions near the gut epithelium where they sometimes interact in highly organized lymphopoietic tissues (Bemark et al., 2024). An overarching feature in these systems is the presence of mechanisms that regulate immune function in an environment replete with microbial signals that, under other circumstances, could lead to an uncontrolled immune response. This specialization at mucosal surfaces extends to other classes of jawed vertebrates, sometimes involving orthologous systems, but often using convergent mechanisms (e.g (Danilova et al., 2005; Flowers et al., 2021; Hansen et al., 2005; Mussmann et al., 1996)). Thus, even among the jawed vertebrates there appear to be diverse, and sometimes convergent, solutions to B cell function at mucosal surfaces. In agnathans, the front-line mechanisms of mucosal immunity will likely be convergent or fundamentally different from those of jawed vertebrates, given the divergent structure of their diversified receptors and the differences in VLRB cell gene networks. These differences are compounded by the long divergence time and distinct genomic histories of extant gnathostome and agnathan lineages. What this means is that we can use what is known in jawed vertebrates as a source of general principles and evolutionary drivers of gut-associated immunity, but we should be open to the presence of mechanisms that depart from those of Ig-based systems in jawed vertebrates.

In sea lamprey ammocoete larvae, both the ventral region of the nephric fold and the gut-associated typhlosole are major sites of VLRB gene assembly and lymphopoiesis (Boehm et al., 2018). As lampreys metamorphose, the typhlosole disappears as part of a major restructuring of the gut (Youson and Horbert, 1982). Lymphopoiesis then moves to the region above the spinal cord known as the supraneural body (also known as the protovertebral arch or dorsal fat body) (Ardavin et al., 1984). This coincides initially with a temporary cessation of feeding and after migration to open water, a radical change in feeding mode from filter feeding to predation on fish body fluids.

**Figure 2:**
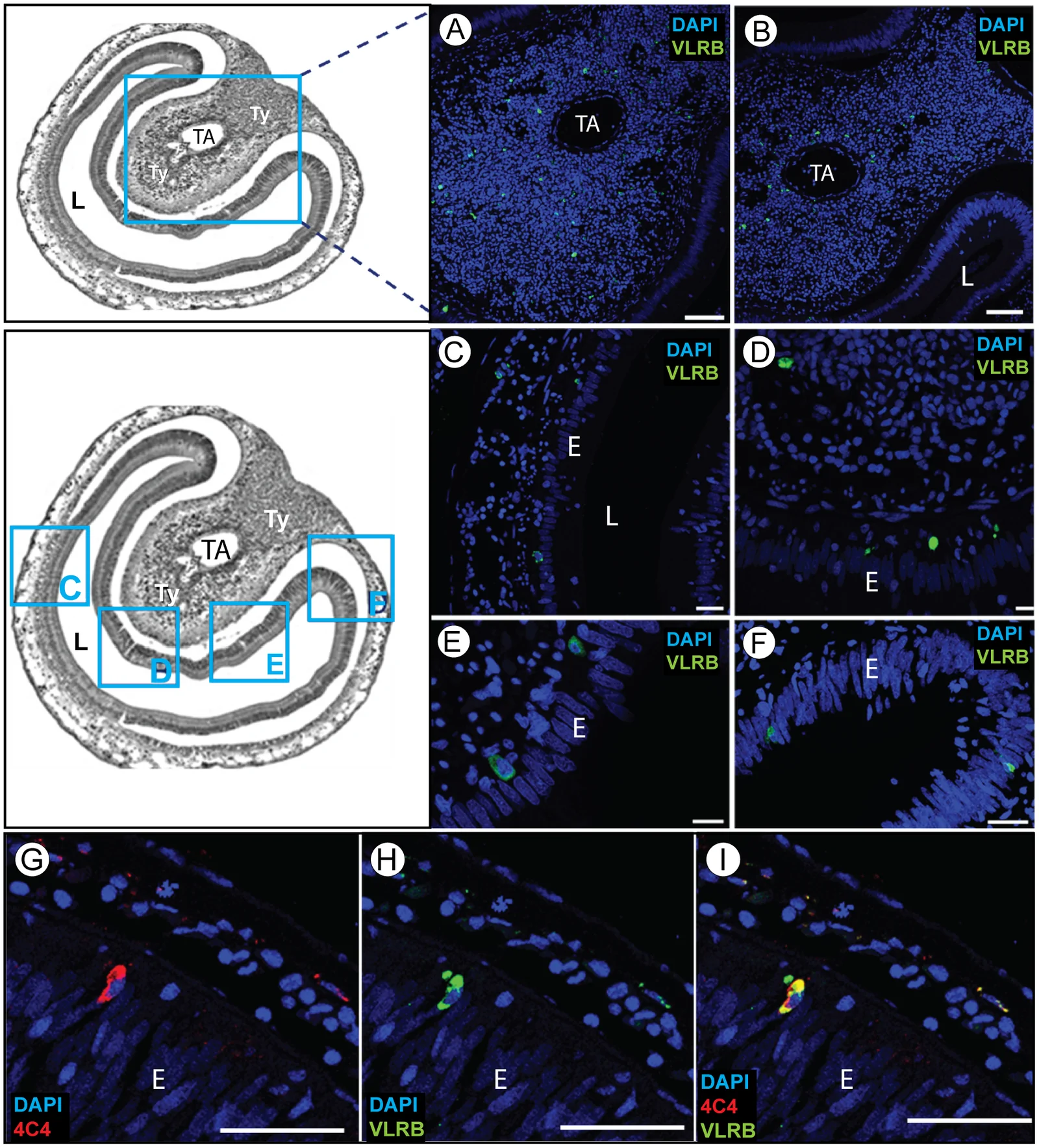
VLRB expression in the gut and typhlosole. Hybridization chain reaction (HCR) *in situ* hybridization images of VLRB expression are oriented relative to a cross-section of the intestine and typhlosole shown on the right. **(A, B)** VLRB^+^ cells within the typhlosole **(A)** and surrounding the typhlosolar artery **(B)**. **(C–E)** VLRB^+^ cells adjacent to the basal lamina of the columnar gut epithelium. **(F)** VLRB^+^ cells in the apical region of the gut epithelium. **(G–I)** A VLRB^+^ cell among the intestinal epithelial cells. Anti-VLRB mouse monoclonal antibody staining (G; clone 4C4), HCR *in situ* hybridization staining **(H)**, and the merged image **(I)**. L, gut lumen; TA, typhlosolar artery; Ty, typhlosole; E, intestinal epithelium. Methods for HCR ISH and immunohistochemistry (IHC) were performed as previously described (Das et al., 2025, 2023).

Importantly, the lamprey ammocoete larval stage encompasses the majority of the sea lamprey life cycle (three to more than five years), as opposed to the shorter-lived adult stage of one to two years. Thus, unlike some very short animal larval stages, the ammocoete stage is associated with a well-developed functional adaptive immune system. The typhlosole is organized around the typhlosolar artery at the center of a lymphocyte-rich parenchymal region enveloped by the C-shaped cross section of the gut epithelium and lumen (**Figure 2**). Blood enters the intestine through the typhlosolar artery, exits into the typhlosole through small dorsal arterioles, then flows through the spongy hematopoietic tissue of the typhlosole leaving ventrally and entering a plexus of venous channels in the intestinal wall (Percy and Potter, 1979). Thus the typhlosolar cells are in direct contact with the ammocoete circulating blood system. VLRB^+^ cells are found throughout the typhlosole and surrounding the typhlosolar artery (**Figures 2A,B**), consistent with previous studies (Das et al., 2023; Hirano et al., 2013). Their intimate association with mucosal tissues, particularly just beneath the epithelial basal lamina (**Figures 2C–E**) as well as more apically among the epithelial cells and in contact with the mucosa (**Figures 2F–I**), open a set of questions regarding their functions in regulating immunity at these sites. Interestingly, VLRB^+^ cells are often found just beneath the intestinal epithelium. Whether the typhlosole and the VLRB cells in contact with the gut epithelial cells function in an organized process of secondary diversification and selection such as in Peyer’s patches of mammals (Komban et al., 2019; Reboldi and Cyster, 2016), or primary diversification as in the Bursa of Fabricius in birds (Cooper et al., 1965), or in other gut-associated immune functions, such as the IgM and IgT B cells of teleosts (Ding et al., 2025; Salinas et al., 2021; Xu et al., 2020), remains to be determined. While lamprey immune organs and cell structures are very different from those of jawed vertebrates, B-like cells in the lamina propria may serve parallel roles in maintaining mucosal immunity. This raises the possibility that lampreys possess a functional analog to the adaptive mucosal immune system seen in jawed vertebrates.

## Questions to guide further exploration

Our orientation for understanding lamprey mucosal immunity is mainly set by analogy to jawed vertebrates. This is a starting point but the receptor systems are structurally unrelated and the two groups are anciently diverged. Nonetheless, convergence with jawed vertebrate mechanisms could highlight general principles or deep regulatory connections that drive the evolution of adaptive immunity at the gut epithelium. Given what we know about VLRB and sea lamprey B-like cells, several questions arise that are listed below.

1. *How much does convergent evolution contribute to similarities between lamprey B-like and jawed vertebrate B cells?* While cell function and morphology show striking parallels to gnathostome B-like cells, gene expression relationships among agnathan and gnathostome cells are obscured by different histories of genome wide duplication and divergence in regulatory gene expression. This complicates inferences of the ancestral state of these lymphocytes in the common ancestor of extant vertebrates. More complete knowledge of the state of hagfish B-like cells, as well as those of divergent jawed vertebrates such as chondrichthyans, will lend a more solid basis to triangulate an ancestral state.
2. *Are VLRBs secreted into the gut lumen or at the mucosa?* Immunohistochemistry and HCR *in situ* hybridization indicate that large, potentially antibody secreting VLRB cells (a subset of VLRB^+^ cells that stain very strongly for VLRB protein or mRNA) are present within the apical region of the gut epithelium (**Figure 2**). These cells could secrete VLRB antibody into the mucosal layer and gut lumen. Studies using mouse anti-VLRB monoclonal antibodies (Guo et al., 2009) and mass spectrometry studies would shed light on this subject. In ammocoete larvae, these investigations need to take particular care not to contaminate the luminal contents during sample processing especially in the anterior intestine where the typhlosole is present.
3. *How does the gut associated immune system vary with life stage?* Most investigations are done with ammocoete larvae which are small particles filter feeders that ingest on a complex mixture of phytoplankton, microbes, and detritus. After metamorphosis and migration to the sea or larger lakes, the sea lamprey feeds on fish blood and body fluids, a drastic alteration of diet. In general, the most readily available adult lampreys are spawning-stage adults which are non-feeding with degenerate intestines. Feeding parasitic adults with more robust guts are sometimes available and these would be interesting to study also in this regard.
4. *Are there donor cassettes that are specialized to mucosal immunity?* VLRs have no direct analog of constant region isotypes but they do have sequence segments that could in principle tag subtypes to different functions. As a cassette class, the 5′-LRRCT donor cassettes offer the most extensive surface area for possible interactions with specific functional adaptors but sequence in any of the donor cassettes could also serve this purpose. Analysis of VLRB repertoires in localized microenvironments isolated with laser microdissection may provide a pathway into this possibility and could reveal potential regional specialization of donor cassette usage.
5. *Are there analogs of Fc receptors that can use VLRBs as adaptors or act as transporters?* Given the LRR structure of VLRB antibodies, we may expect that any analogs to antibody receptors will be structurally distinct from gnathostome FcRs.
6. *Why are most 5′-LRRCT donor cassettes encoded in clusters that are distant from the VLRB germline gene and other cassette types?* Three of the five VLRB donor cassette clusters encode primarily or entirely 5′-LRRCT cassettes at distances that are megabases away from the germline gene or on separate chromosomes. Are these clustered differentially regulated in the gut (or elsewhere)? Could they have specialized functions?
7. *Why are there at least two regions where CDA2 is highly expressed, correlating with VLRB assembly in the ventral kidney fold and typhlosole?* Given the typhlosole’s intimate proximity to the gut epithelium/lumen, this could indicate a specialized region of VLRB maturation with input from the surrounding immunoregulatory and microbial state of the gut. Detailed analyses of B-like cell state and VLRB repertoire development in response to different paths of immune challenge may illuminate this problem.
8. *Are there secondary diversification events in the lamprey analogous to somatic mutation in jawed vertebrates?* So far there is no evidence for somatic mutation-like maturation of assembled VLRB but the matter is far from thoroughly explored. Affinity maturation could be based on point mutations or some form of secondary gene conversion. Other forms of selected secondary mutagenesis are also possible.
9. *What is the state of mucosal immunity in the hagfish?* Lamprey adaptive immunity differs greatly from that of jawed vertebrates and it is often hard to know which characters are ancient and which are later innovations within the lamprey lineage. The Northern Hemisphere lampreys, which have been studied most extensively with respect to VLRs, diverged from one another relatively recently (10–40 MYA), whereas their lineage diverged from that of the Southern Hemisphere lampreys approximately 170 MYA (Kuraku and Kuratani, 2006). Because hagfish diverged from the lineage leading to the lampreys well before the last common ancestor of the modern lampreys (~450 MYA), common features can be used to infer early and essential features of the agnathan immune system.
10. *Why is the typhlosole, a primary site of lamprey VLRB assembly, so closely associated with the gut epithelium and does this intimate association implicate gut immunity and microbial activity in the gut as a primary evolutionary driver of adaptive immunity in the lampreys?* Specialization in regulatory programs of gut associated sea lamprey B-like cells, even if it differs from that of gnathostomes, may be evident in scRNA-seq studies of intestinal lymphocytes supplemented by HCR *in situ* hybridization of candidate differentially expressed genes. Laser capture microscopy and spatial genomics analyses of the gut epithelium and typhlosole will also help in these studies. Analysis of VLRB repertoires in the typhlosole and the potential effects of microbiome perturbations on VLRB gene assembly and repertoire will be critical to understanding this system. Even if receptor-proximate mechanisms prove to be convergent, fundamental regulatory mechanisms to control immune response in the intestinal environment may be distantly shared.

## Conclusions

From a developmental perspective, assembled VLRB genes are first detectable in sea lamprey coincident with the onset of filter feeding at about 30 days of development. The intimate connection of the gut epithelium to the tissues where assembly of at least a major portion of mature VLRB sequences takes place begs the question of how VLRB antibodies and VLRB-bearing B-like cells may function at the gut epithelium and lumen. It also suggests that information about the state of the gut lumen may affect VLRB assembly as well as B-like cell gene expression and development. While lamprey B-like cells have similarities with gnathostome B cells in both morphology and gene expression, they are also quite different beyond the nature of their diversified receptors. Given these differences, and the coincidence of the agnathans-gnathostome divergence with the evolutionary emergence of vertebrate adaptive immunity, study of sea lamprey B-like cells will pinpoint fundamental mechanisms of vertebrate adaptive immunity including the role of B-like cells in the modulation of microbial activity in the gut.

## Data Availability

The raw data supporting the conclusions of this article will be made available by the authors, without undue reservation.
